# Interventions to cultivate physician empathy: a systematic review

**DOI:** 10.1186/1472-6920-14-219

**Published:** 2014-10-14

**Authors:** Zak Kelm, James Womer, Jennifer K Walter, Chris Feudtner

**Affiliations:** Ohio University Heritage College of Osteopathic Medicine, Dublin, OH USA; Temple University School of Medicine, Philadelphia, PA USA; Department of Medical Ethics, The Children’s Hospital of Philadelphia, Philadelphia, PA USA

**Keywords:** Empathy, Compassion emotional intelligence, Undergraduate medical education, Graduate medical education, Continuing medical education, Internship and residency

## Abstract

**Background:**

Physician empathy is both theoretically and empirically critical to patient health, but research indicates that empathy declines throughout medical school and is lower than ideal among physicians. In this paper, we synthesize the published literature regarding interventions that were quantitatively evaluated to detect changes in empathy among medical students, residents, fellows and physicians.

**Methods:**

We systematically searched PubMed, EMBASE, Web of Science and PsychINFO in June of 2014 to identify articles that quantitatively assessed changes in empathy due to interventions among medical students, residents, fellows and physicians.

**Results:**

Of the 1,415 articles identified, 64 met inclusion criteria. We qualitatively synthesized the findings of qualified studies by extracting data for ten study metrics: 1) source population, 2) sample size, 3) control group, 4) random assignment, 5) intervention type, 6) intervention duration, 7) assessment strategy, 8) type of outcome measure, 9) outcome assessment time frame, and 10) whether a statistically significant increase in empathy was reported. Overall, the 64 included studies were characterized by relatively poor research designs, insufficient reporting of intervention procedures, low incidence of patient-report empathy assessment measures, and inadequate evaluations of long-term efficacy. 8 of 10 studies with highly rigorous designs, however, found that targeted interventions did increase empathy.

**Conclusions:**

Physician empathy appears to be an important aspect of patient and physician well-being. Although the current empathy intervention literature is limited by a variety of methodological weaknesses, a sample of high-quality study designs provides initial support for the notion that physician empathy can be enhanced through interventions. Future research should strive to increase the sample of high-quality designs through more randomized, controlled studies with valid measures, explicit reporting of intervention strategies and procedures, and long-term efficacy assessments.

**Electronic supplementary material:**

The online version of this article (doi:10.1186/1472-6920-14-219) contains supplementary material, which is available to authorized users.

## Background

In their Learning Objectives for Medical School Education, the Association of American Medical Colleges states that, “physicians must be compassionate and empathetic in caring for patients” [1]. Similarly, the American Medical Association’s first principle of medical ethics asserts the following: “A physician shall be dedicated to providing competent medical care, with compassion and respect for human dignity and rights” [2]. These statements illustrate that the field of medicine is not only committed to producing and upholding the most knowledgeable and skillful physicians possible, but also the most caring and empathic. Within the field of medicine, there is disagreement regarding the precise definition of empathy [3, 4]. Some researchers define physician empathy as a “cognitive attribute that involves an ability to understand the patient’s inner experiences and perspective and a capability to communicate this understanding” [3]. Others describe four components of the empathy construct: 1) emotive, the ability to imagine and share a patient’s psychological state or feelings; 2) moral, the physician’s internal motivation to express empathy; 3) cognitive, the intellectual ability to identify and understand a patient’s perspectives and emotions; and 4) behavioral, the ability to communicate this understanding of the patient’s perspectives and emotions [5]. Most constructions of empathy have in common, however, an understanding of the emotional states of others and expression of this understanding.

While there is some disagreement regarding the exact components of empathy, there is wide consensus that physician empathy significantly affects patients in a variety of ways. Physician empathy has been associated with higher levels of patient satisfaction [6–12], adherence to medical recommendations or regimens [10, 13–16], and improved clinical outcomes [6, 16–20]. Moreover, empathy appears to positively influence physicians themselves, as empathy has been linked to lower burnout [21], higher well-being [21–23], higher ratings of clinical competence [3], and less medical-legal risk [24–26]. Physician empathy may even reduce health care costs, as patient centered communication styles have been associated with lower diagnostic test expenditures [27].

Despite considerable evidence demonstrating the benefits of physician empathy for patients and physicians, empathy is at a lower than ideal level in medicine. Studies indicate that physicians often overlook or miss empathic opportunities during patient encounters [28–32], and tend to spend significantly more time and energy on biomedical inquiry and offering medical explanations to patients [8, 32]. In one study, physicians acknowledged or explored empathic opportunities only 10% of the time [32]. Patient reports also point to a shortage of physician empathy [33]. Yet, not only is there a shortage of empathy among medical students and physicians, numerous studies show that empathy declines throughout medical training, in both medical school and residency [34–39]. As trainees experience an increase in personal distress from burnout, higher rates of depression and decreased quality of life during their training, they are less likely to experience or demonstrate empathy. This distress is potentially promoted by deficiencies in several aspects of the medical curricula, including the formal (e.g. lack of formal empathy training), informal (e.g. inadequate mentors, shorter hospital stays, and inappropriate learning environments), and hidden (e.g. mistreatment of students and high workload) medical curricula [38].

The lack of empathy among physicians and the decline in empathy throughout medical training offer reasons for concern, especially given the relationship between physician empathy and patient health and well-being [6–20]. It is incumbent upon medical educators, and the field in general, to investigate methods to enhance medical student and physician empathy. Although studies have reviewed and examined interventions to increase empathy among medical students [40] and in health and human services [41], no review has been done on the full body of literature regarding interventions designed to quantitatively detect changes in medical student or physician empathy. Thus, the present study seeks to systematically review and synthesize the existing literature of quantitatively evaluated interventions aimed at cultivating empathy among medical students, residents, fellows, and attending physicians.

## Methods

**Figure 1:**
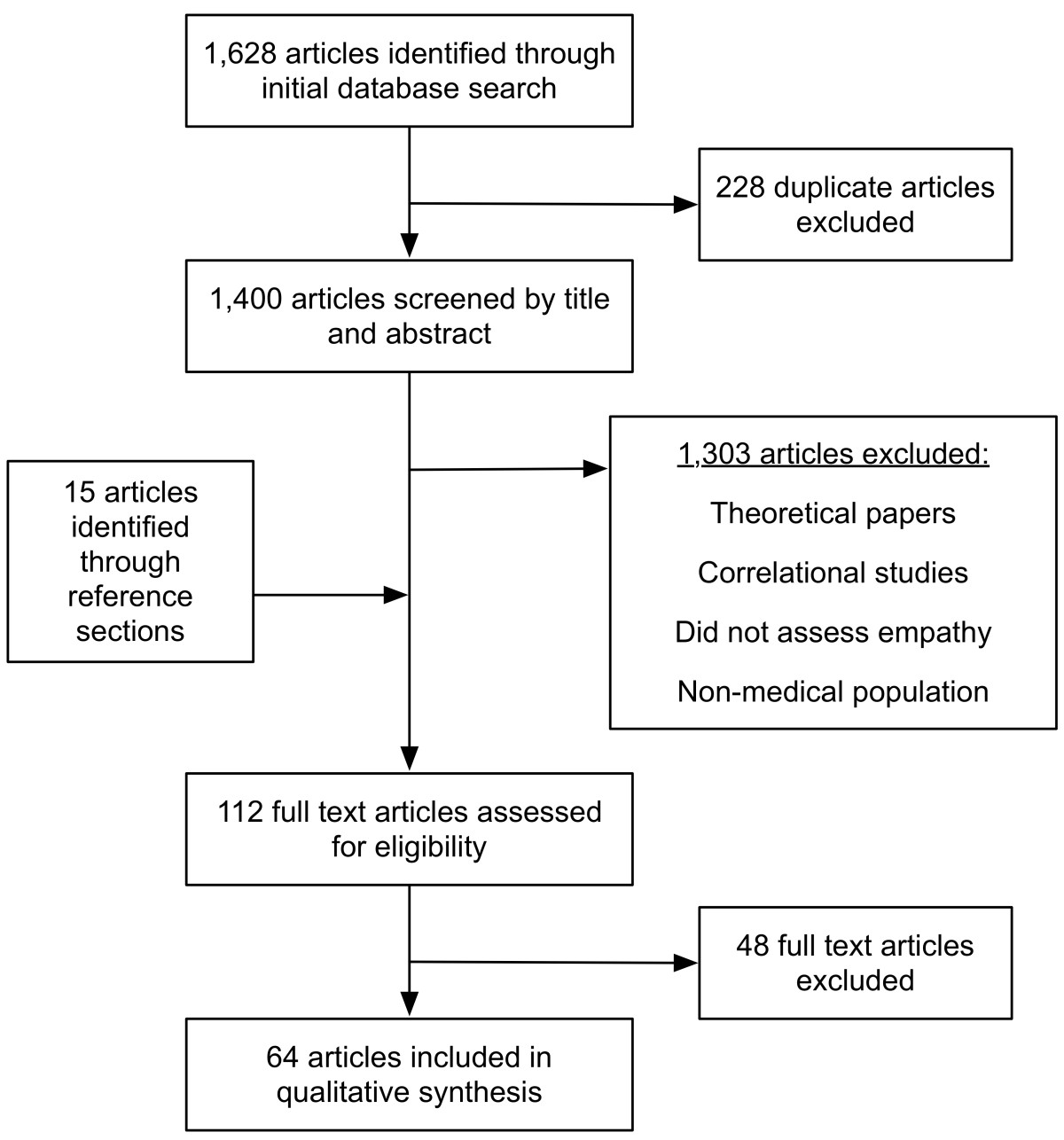
**Study flow diagram.** Illustration of database search process to identify studies that met inclusion criteria.

In June of 2014, we conducted a systematic review of the literature, searching the online databases PubMed, EMBASE, Web of Science, and PsychINFO (Figure 1). We collaborated with a librarian from the Biomedical Library at the University of Pennsylvania to discuss and refine our search strategy.

In PubMed, we used the following search terms as key words: (1) empathy or caring or compassion, (2) medical students or physicians, (3) medical education or clinical competence or training or workshop, and (4) communication. This search generated 574 articles. In EMBASE, the following search terms were used as descriptors: (1) empathy, (2) medical student or resident or physician, (3) medical education, (4) clinical competence, and (5) doctor patient relation or interpersonal communication. This search produced 359 articles. In Web of Science, we searched using the following key terms: (1) empathy, (2) medical students or physicians, and (3) education or training or workshop or intervention. This search generated 550 articles. Finally, in PsychINFO, we used the following descriptors: (1) empathy, (2) medical students or physicians, and (3) medical education or training. The PsychINFO search produced 145 articles.

Overall, our initial literature search generated 1,628 citations. Elimination of duplicate articles produced 1,400 citations, and an additional 15 articles were identified through references. In turn, 1,415 articles were screened by title and abstract. Of these, 1,303 articles were excluded from further review, including correlational studies, theoretical papers, articles that failed to assess empathy, and publications with non-medical populations. The full texts of the remaining 112 studies were retrieved and assessed for eligibility using the inclusion criteria outlined below, yielding 64 eligible studies.

Our inclusion criteria specified that each study: 1) elucidate that some type of intervention was used, 2) explicitly state that “empathy” was being evaluated or measured, 3) assess changes in empathy by reporting quantitative outcomes using statistical methods, and 4) examine empathy in medical students, residents, fellows or physicians. We considered only articles written in the English language. After reviewing the titles and abstracts of all articles retrieved through our initial database search, we obtained full texts of potentially eligible studies. We also reviewed the reference sections of these selected articles to obtain any additional studies not found through our initial database search.

Members of the research team (ZK, JW) independently examined the full texts of studies that passed title and abstract review and met inclusion criteria. Each article was assessed for a variety of metrics, including source population, sample size, type of intervention, duration of intervention, assessment strategy, type of outcome measure, and outcome assessment time frame. Data were extracted and finalized through discussion among the research team.

The review adhered to the Preferred Reporting Items for Systematic Reviews and Meta-Analyses (PRISMA) guidelines (see Additional file 1).

## Results

We identified 64 studies [42–105] that quantitatively assessed empathy interventions in our specified population (see Additional file 2). 50 of these 64 studies were published within the past ten years. We reviewed the findings from these articles by extracting data for ten major study metrics: 1) the study *source population*, 2) the study *sample size*, 3) the presence or absence of a *control group*, 4) whether or not *random assignment* was used, 5) the *type of intervention*, 6) the *duration of the intervention*, 7) the empathy *assessment strategy*, 8) the *type of outcome measure*, 9) the *outcome assessment time frame* post-intervention, and 10) whether or not a statistically *significant increase in empathy* was reported. This summary method allowed the research team to investigate similarities and differences among articles and to attain a more general appreciation for the strengths and weaknesses of the current literature regarding empathy interventions. Thus, the results are presented and organized around the ten metrics used to evaluate each study.

1. Source population
  The source population assessment was divided into four major categories: medical students, residents, fellows and physicians. Of the 64 studies reviewed, 36 (56%) evaluated empathy interventions with medical students, 13 (20%) with residents, 2 (3%) with fellows, and 15 (23%) with attending physicians. In addition, six studies evaluated empathy in a “mixed” population, including three articles assessing both medical students and residents, two assessing residents and fellows, and one article assessing medical students and attending physicians.
2. Sample size
  Sample sizes ranged from 11 participants to 439 participants. The mean sample size was approximately 89 participants, with a median of 78, Q1 of 28, Q3 of 125, and a standard deviation of 75. 21 studies (33%) exhibited sample sizes of 100 or more participants, while 24 studies (38%) reported sample sizes of fewer than 50 participants.
3. Control group
  Of the 64 studies reviewed, 35 (55%) used a control or comparison group.
4. Random assignment
  Of the 35 controlled interventions, 24 (69%) used random assignment.
5. Intervention type
  A variety of intervention types were utilized. 20 studies (31%) employed “communication skills training” interventions. We classified an intervention as “communication skills training” if the study authors explicitly referred to their intervention as a communication skills training or workshop. Often, communication skills training interventions were comprised of a variety of features, including didactic sessions on effective communication and empathy, experiential learning, and skills or behavior-based workshops. For instance, Winefield and Chur-Hansen [103] used both didactic material (i.e., lecture, videotape and handouts) and training workshops in which medical students practiced their communications skills by interviewing standardized patients and receiving feedback. Tulsky et al. [99] used an audiotape CD-Rom training program that allowed physicians to observe the demonstration of an effective communication skill and review or reflect upon their own conversations and implementation of the skill.
  Seven studies (11%) primarily used a “role playing” intervention, typically involving experiential learning in which study participants acted as a patient or family member. For example, Chunharas et al. [51] sought to build medical student empathy for patients receiving intramuscular or subcutaneous injection by asking medical students to take turns injecting each other with saline solution.
  Six studies (9%) utilized some form of the “humanities,” including reflective writing, a literature course, and theater. For instance, Shapiro et al. [93] used a reflective writing intervention, in which medical students wrote essays from the point of view of either hypothetical or standardized patients.
  In addition, two articles reported an intervention involving “motivational interviewing training,” a counseling approach aimed at patient behavior change. Three interventions used “balint training,” which entails small group discussions focused on patient emotions. Two studies emphasized mindfulness-based stress reduction (MBSR), a type of meditation characterized by nonjudgmental, moment-to-moment awareness. One intervention used problem-based learning sessions that focused on empathy and communication.
  Finally, 23 studies (35%) were categorized as “other.” Those studies classified as “other” could not be logically organized into a more general category. Studies classified as “other” often used a variety of intervention types. For instance, Krasner et al. [69] constructed an intervention that involved MBSR and the humanities, particularly reflective writing, appreciative inquiry exercises, and other educational and experiential tasks. Riess et al. [82] created an empathy training protocol that included education in the neurobiology and physiology of empathy, real-time biofeedback during physician-patient encounters, and mindfulness exercises. In addition, many of the “other” type interventions exhibited similarities to “communication skills training” interventions, as various didactic, experiential or skills-based elements were utilized.
6. Duration of intervention
  Duration of empathy interventions (i.e., amount of time spent on intervention activities) ranged from 40 minutes to approximately 96 hours, with a mean of 15 hours, median 12 hours, Q1 of 4 hours, Q3 of 18 hours, and a standard deviation of 74.4 hours. Interventions occurred over the course of days, weeks, months, and even years. 19 studies (30%) were regarded as “not explicit” (N/E) in their reporting of intervention duration, particularly the number of intervention hours.
7. Assessment strategy
  Empathy assessment strategies were evaluated using two major categories: timing of the empathy assessment (pre- versus post-intervention) and overall study design evaluation (within-group versus between-group). 58 (91%) of the 64 studies assessed empathy both pre- and post-intervention. 15 studies (23%) used between-group comparison to evaluate empathy changes, 32 studies (50%) used within-group comparison methods, and 17 (27%) utilized both a within- and between-group assessment strategy.
8. Outcome measures
  Among the outcome measures used to assess changes in empathy, 31 studies (48%) used self-report measures. 33 (52%) employed other-report measures where others (including patients) evaluated their perception of a medical practitioner’s empathy. Only six studies (9%) evaluated changes in empathy using patient reports. Four assessed empathy using more than one type of outcome measure.
  Self-report measures involved a self-report survey or single question. A variety of self-report survey types were used, including the Jefferson Scale of Physician Empathy (JSPE), Empathic Tendency Scale (ETS), Empathic Skill Scale (ESS), Balanced Emotional Empathy Scale (BEES), Empathy Construct Rating Scale (ECRS), and Interpersonal Reactivity Index (IRI). The JSPE was the most commonly used self-report survey, appearing in 15 of the 31 studies employing self-report measures.
  Other-report outcome measures varied as well. Typically, these measures involved assessments of participant behaviors during clinical encounters or medical interviews by trained observers. For instance, Bonvicini et al. [46] used trained observers and an empathy coding system to evaluate physician empathy during audiotaped recordings of physician-patient interactions. 24 of the 32 articles employing other-report measures evaluated empathy during real or staged patient encounters. Six studies assessed empathy based on medical students’ or residents’ written responses to hypothetical patient scenarios, and two studies used tests requiring decoding of emotional facial expressions.
9. Outcome assessment time frame
  Outcome assessment time frames ranged from immediately following the intervention to 21 years post-intervention. We defined immediately following the intervention as studies that measured empathy within a day after the end of an intervention. 30 studies (47%) assessed empathy immediately following the intervention. 17 studies (27%) evaluated empathy at some time after immediate assessment. Of these studies, seven (11%) assessed empathy 1–4 weeks post-intervention, ten (15%) assessed empathy 1–6 months post-intervention, three evaluated 12 months post-intervention, one study assessed after 3 years, and one study assessed empathy between four and 21 years post-intervention. Nine studies evaluated empathy at multiple time points post-intervention. 20 studies (31%) were not explicit (N/E) about the outcome assessment time frame.
10. Significant increase in empathy reported
  42 (66%) of the 64 reviewed studies reported a statistically significant increase in empathy. 14 studies (22%) showed no significant change in empathy. Finally, eight studies (12%) were classified as “mixed” because they reported some measure with no significant change in empathy and another measure with a significant increase in empathy. Cahan et al. [49] reported the results of two distinct pilot studies. Pilot 1 was a between-group study design that showed no significant result. Pilot 2 used a within-group study design that resulted in a significant increase in empathy. Both Chunharas et al. [51] and Norfolk et al. [76] reported a significant increase in empathy within groups, but no increase resulted from between-group comparison. Riess et al. [81] reported a significant increase in empathy on the Consultation and Relational Empathy (CARE) measure, but no change on the Balanced Emotional Empathy Scale (BEES), Jefferson Scale of Physician Empathy (JSPE), and Ekman Facial Decoding Test. Riess et al. [82] found a significant increase in empathy on the CARE measure, the Neurobiology and Physiology of Empathy Test and the Ekman Facial Decoding test, but no significant increase on the BEES and JSPE. Sanson-Fisher & Poole [87] reported no significant change in empathy when evaluated within the intervention group, but a significant increase resulted from between-group comparison. Shapiro et al. [92] found an increase in empathy on the BEES, but no change on the Empathy Construct Rating Scale (ECRS). Finally, Shapiro et al. [93] used a study-specific thematic coding system for writing samples of medical students and found an increase in empathy for physicians, but no change in empathy for the family or patient.

### Study design quality assessment

Given that two thirds of included studies reported a significant increase in empathy (not including mixed results), we performed a qualitative assessment of study design quality (see Additional file 3). Study design quality was based on three metrics: 1) presence or absence of a control group, 2) whether or not random assignment was used, and 3) the reliability and validity of the outcome measure. Further, outcome measures were categorized into three types: 1) reliable and valid outcome measure (+), 2) reliable, but not valid outcome measure (+/−), and 3) neither reliable, nor valid outcome measure (−). If reliability or validity information was not available in published material, efforts were made to contact the authors to obtain this information.

The three quality metrics were used to establish a study design rating system composed of three tiers. 10 studies (16%) were classified as Tier 1. Tier 1 studies were the most rigorous, involving randomized, controlled interventions, along with reliable and valid outcome measures (+). 9 studies (14%) were categorized as Tier 2. Tier 2 studies were composed of one of two quality metric arrangements: 1) randomized, controlled interventions, along with reliable, but not valid outcome measures (+/−), or 2) controlled interventions devoid of random assignment, along with reliable and valid outcome measures (+). Finally, 45 studies (70%) were classified as Tier 3: all other study designs.

**Figure 2:**
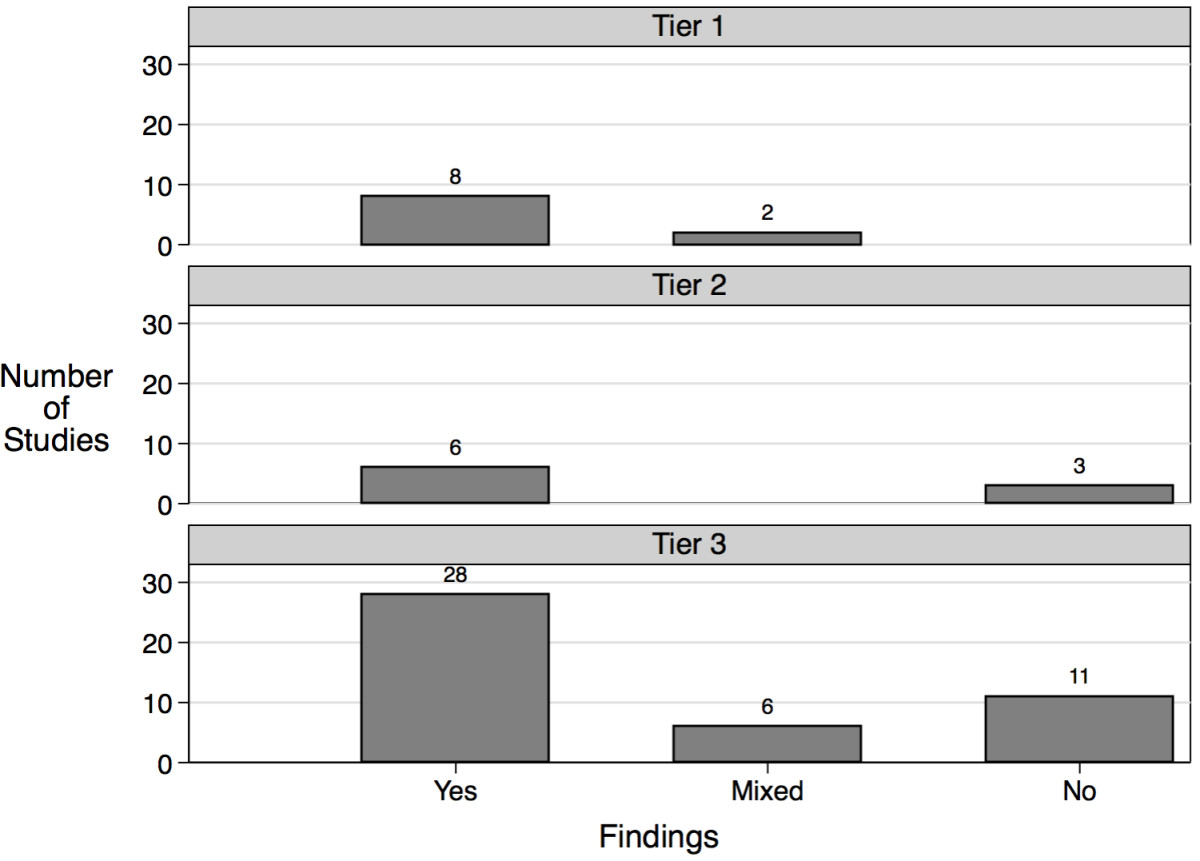
**Comparison of study design quality with significant increases in empathy.** Number of studies exhibiting significant, non-significant and mixed effects among Tiers 1, 2 and 3.

We compared study design quality to our significant increase in empathy metric (Figure 2, Additional file 3). Of the ten studies classified as Tier 1, eight reported a significant increase in empathy, and two showed mixed results. Of the nine studies classified as Tier 2, six (66%) showed a significant increase in empthy and three (33%) exhibited no change in empathy. Of the 45 studies classified as Tier 3, 28 (62%) reported a significant increase in empathy, six (13%) showed mixed results, and eleven (24%) reported no change in empathy. Compared to the 80% of studies showing a significant increase in empathy in Tier 1, 63% (34/54) of studies in Tier 2 and Tier 3 reported a significant increase in empathy. Moreover, all 14 studies that reported no significant change in empathy were categorized into either Tier 2 or Tier 3.

### Characteristics of tier 1 studies

Tier 1 studies were heterogeneous in their source populations: 50% involved medical students and 50% involved residents, fellows, or physicians, suggesting that empathy interventions may be effective during or after training. A variety of intervention types were used in Tier 1 studies: 30% had been classified as “communication skills training,” 40% were categorized as “other,” and “role playing,” “motivational interviewing,” and “humanities” interventions were each represented by a single study. Tier 1 studies also exhibited a relatively balanced array of outcome measure types, as 60% used other-report measures, 50% employed self-report measures, and 30% used patient-report measures. Furthermore, 50% of Tier 1 studies evaluated empathy 1–6 months post-intervention. Five Tier 1 studies reported effect size data; however, no two of those five used the same methodology to calculate effect size, leaving us unable to make meaningful comparisons based on those data.

## Discussion

The present study provides a novel synthesis and analysis of empathy interventions in medicine. Previous studies have systematically reviewed empathy measures and their relationship to patient outcomes in cancer care [106], emotion skills training for medical students [107], and empirical research on empathy in medical students and physicians [108]. This study, however, has systematically reviewed and synthesized interventions that quantitatively evaluate changes in empathy among medical students, residents, fellows, and physicians.

This review has generated a number of key findings. As previously mentioned, 66% of studies reported a significant increase in empathy. While this result was encouraging, we sought to further evaluate this trend by assessment of study design. Results of this assessment indicated that although the majority of studies (84%) lacked highly rigorous study designs (Tier 2 and Tier 3), all ten studies classified as Tier 1 exhibited either significant increases in empathy or mixed effects. Moreover, 80% of Tier 1 studies showed a significant increase in empathy (not mixed), while only 63% of studies in Tier 2 and Tier 3 reported significant increases. Despite the small number of studies, these findings generally support the hypothesis that intervention can increase empathy among physicians and medical students, not only because of the high incidence of significant increases in empathy in Tier 1, but also because Tier 1 studies included balanced assortments of other study metrics (e.g., study population, intervention type, outcome measure type, and outcome assessment time frame). Taken together, the Tier 1 results suggest that empathy can be enhanced through a variety of intervention types targeted toward medical students, residents, fellows and physicians, and that increased empathy may persist beyond the immediate post-intervention period.

Although these findings are encouraging, it is important to highlight the fact that only ten studies were classified as Tier 1. Further, only half of the Tier 1 studies explicitly reported effect sizes. These findings add uncertainty to our inferences about the cultivation of empathy among medical students and physicians, and also point to major limitations associated with the full body of physician empathy intervention literature: significant fractions of eligible studies lacked rigorous study designs, lacked control groups, and failed to use random assignment. Well-controlled and randomized studies are the most reliable way to account for, or minimize, potential confounding factors, and the fact that they are rare in the physician empathy intervention literature should be taken into account when examining the high incidence of significant outcomes. The overall literature was also marked by relatively small sample sizes and vague reporting of intervention durations and outcome-assessment time frames. While empathy interventions were classified into different categories, the literature was characterized by a wide array of intervention types that typically showed both similar and disparate underlying features. In some cases, articles lacked detailed descriptions of the intervention. If the ultimate goal is implementation of effective empathy-increasing interventions, the literature does not enable other institutions to replicate these outcomes.

Outcome assessment time frames, and particularly the high prevalence of studies only assessing empathy immediately following the intervention, should also be highlighted as a weakness of the current literature. While medical student or physician empathy may significantly increase immediately after an intervention, there is limited insight available about long-term efficacy. Just over a quarter of studies explicitly reported follow-up quantitative evaluations of empathy at some time (i.e., 1 week to 21 years) after an immediate assessment of the intervention.

The majority of empathy interventions were targeted toward medical students. Although this trend is not surprising given that medical school has an explicit curriculum, including communication skills training in a growing number of institutions, researchers and educators should be wary of the fact that these empathic skills degrade over time [34–39]. Therefore, interventions aimed at enhancing empathy among residents, fellows and physicians may be more important to ensure that patients consistently receive empathic care from their physicians. Little is known about the long-term efficacy of empathy interventions. Even if medical student empathy is enhanced through interventions, a lack of long-term efficacy could have serious consequences for arguably the most critical population – practicing physicians and their patients.

Another limitation of the literature involved outcome measure type. Empathy was measured in a variety of ways, but the vast majority of studies used self-report or other-report measures, and only six employed patient reports to measure physician empathy. Indeed, close to half of the reviewed studies used self-report measures to evaluate changes in empathy. Some of these self-report surveys are psychometrically reliable and validated, yet little is known about the relationship between self-report measures of empathy and behavioral or patient-report measures. It is also the case that measurements of empathy in a medical population may be subject to significant social desirability bias; therefore, particularly with self-report measures of empathy, it can be difficult to say whether interventions increase empathy, or awareness of the desirability of an empathetic physician. Self-report surveys can be an effective and reliable measure of physician empathy, but they must be validated against behavioral or patient-report measures.

A recent study indicated that a commonly used self-report measure, the Jefferson Scale of Physician Empathy (JSPE) [109–111], exhibited statistically significant correlations with a patient-report measure, the Jefferson Scale of Patient Perceptions of Physician Empathy (JSPPPE) [112]. The strongest correlations have been demonstrated with real patients [113], although weaker correlations exist with standardized patients as well [112]. Further, a systematic review concluded that physician empathy is associated with beneficial outcomes based on patient-report measures in cancer care. However, little is known about the relationship between the reliable and valid patient-report measures examined in the cancer care study and physician self-report empathy measures like the JSPE [106]. In other words, there may be a misalignment between the outcome measure type (patient-report) most prevalent in studies investigating the association between physician empathy and patient outcomes, and an outcome measure type common to empathy interventions (self-report). It is worth noting that while only six studies overall used patient-report measures, three of these studies were categorized as Tier 1. Further, all three of these Tier 1 studies employed reliable and valid patient-report measures that have been associated with beneficial patient outcomes. These results may add confidence to the inference that targeted interventions may not only increase empathy, but also lead to beneficial effects for patients.

In addition to the weaknesses of the current empathy intervention literature, our study may have been marked by a number of limitations, such as that imposed by the availability of information in the published materials (e.g., intervention durations, procedures, outcome assessment time frames, and reliability and validity of measures). Our efforts to contact authors to obtain further information yielded mixed results. Our study may have also been limited by a ‘publication bias’, which could relate to the high incidence of studies reporting significant effects. Thus, the literature may contain a disproportionately small number of null results.

Overall, results of the study design quality assessment suggest that empathy can be enhanced in our study population. However, given the relatively small number of Tier 1 studies and limitations of the full body of literature, we suggest strategies to facilitate progress within empathy interventions for medical students, residents, fellows, and physicians: 1) Further determining the correlation between self-report, other-report (behavioral), and patient-report measures of physician empathy to ensure future studies are able to utilize reliable and validated measures that have an established connection between change in self-report and increase in patient perception of empathy; 2) Establishing consensus about which measurement types should be used to evaluate physician empathy so that smaller studies may be aggregated in the future in a meta-analysis; 3) Ensuring adequate and explicit reporting of intervention procedures and implementation to promote transparent and easily replicable studies; 4) Conducting more high-quality randomized controlled study designs to establish a larger sample of Tier 1 studies, and thereby evaluating their efficacy with a higher degree of confidence because confounding factors have been controlled for; 5) Given the degradation of empathy, recognizing the need to develop and test interventions at multiple time points in training and practice of medicine; and 6) Lengthening outcome assessment time frames to investigate the long-term efficacy of empathy interventions.

## Conclusions

Although considerably more research must be undertaken, the present study provides valuable insight into the current state of the empathy intervention literature and suggests that targeted interventions may be able to cultivate physician empathy. The reported shortage of empathy and decline in empathy during medical training only amplifies the importance of finding reliable interventions for physicians and physicians-in-training. Indeed, heightened empathy among medical practitioners could not only lead to a more ethical healthcare system, but also to enhanced health and well-being for patients and practitioners themselves.

## Electronic supplementary material

Additional file 1: PRISMA 2009 Checklist. Table reporting key PRISMA items by section and page number of the systematic review. (DOC 62 KB)

Additional file 2: Table S1: Intervention studies evaluating quantitative changes in empathy. Table showing the results of all data extraction measures for the 64 qualifying studies. (DOCX 146 KB)

Additional file 3: Table S2: Quality assessment of intervention studies. Description of Dataset: Table showing the quality assessment results of all 64 studies. (DOCX 114 KB)

Below are the links to the authors’ original submitted files for images.

Authors’ original file for figure 1

Authors’ original file for figure 2
