# Supplementary material for: Interventions to cultivate physician empathy: a systematic review
Source: BMC Med Educ. 2014 Oct 14;14:219. doi: 10.1186/1472-6920-14-219 (PMC4201694; doi:10.1186/1472-6920-14-219)
Supplement: Supplementary file 2 — Additional file 2: Table S1: Intervention studies evaluating quantitative changes in empathy. Table showing the results of all data extraction measures for the 64 qualifying studies. (DOCX 146 KB) [file 12909_2013_1041_MOESM2_ESM.docx]

**Table 1. Intervention studies evaluating quantitative changes in empathy.**

| **Article** | **Source Population** | **Sample Size** | **Control Group** | **Random Assignment** | **Intervention Type** | **Duration of Intervention (hours)** | **Assessment Strategy (pre/post; within/between)** | **Type of Outcome Measure** | **Outcome Assessment Time Frame** | **Sig Increase in Empathy?** |
| --- | --- | --- | --- | --- | --- | --- | --- | --- | --- | --- |
| Airagnes et al,. 2014 [42] | Medical Students | 439 | Yes | No | Balint Training | 20 | Pre & post; within & between-group | Self-report survey | Immediately following intervention | No |
| Bayne, 2011 [43] | Medical Students | 22 | No | N/A | Other | 4 | Pre & post; within-group | Patient-report survey | 1 week post-intervention | Yes |
| Bays et al., 2014 [44] | Residents & Fellows | 128 | No | No | Communication Skills Training | 32 | Pre & post; within-group | Other-report | Immediately following intervention | Yes |
| Bond et al., 2013 [45] | Medical Students | 27 | No | N/A | Mindfulness Based Stress Reduction (MBSR) | 16.5 | Pre & post; within-group | Self-report survey | 1 week post-intervention | No |
| Bonvicini et al., 2009 [46] | Physicians | 114 | Yes | Yes | Communication Skills Training | 18 | Pre & post; within & between-group | Other-report | 6 months post-intervention | Yes |
| Bosse et al., 2012 [47] | Medical Students | 92 | Yes | Yes | Communication Skills Training | N/E | Post; between-group | Other-report | N/E | Yes |
| Bunn & Terpstra, 2009 [48] | Medical Students | 150 | Yes | Yes | Role Playing | 0.67 (40 minutes) | Pre & post; within & between-group | Self-report survey | Immediately following intervention | Yes |
| Cahan et al., 2010 [49] | Medical Students | 97 (pilot 1), 44 (pilot 2) | Yes (pilot 1), No (pilot 2) | No (pilot 1), N/A (pilot 2) | Other | N/E | Post; between-group (1), pre & post; within-group (2) | Other- report | N/E | Mixed |
| Cataldo et al., 2005 [50] | Medical Students & Physicians | 114 | Yes | No | Balint Training | 96 | Post; between-group | Self-report survey | 4-21 years post-intervention | No |
| Chunharas et al., 2013 [51] | Medical Students | 89 | Yes | Yes | Role Playing | N/E | Pre & post; within & between-group | Self-report question | Immediately following intervention | Mixed |
| Cinar et al., 2012 [52] | Residents | 20 | No | N/A | Communication Skills Training | 9 | Pre & post; within-group | Self-report survey | N/E | No |

N/A, Not Applicable; N/E, Not Explicitly Stated

**Table 1. Intervention studies evaluating quantitative changes in empathy.**

| **Article** | **Source Population** | **Sample Size** | **Control Group** | **Random Assignment** | **Intervention Type** | **Duration of Intervention (hours)** | **Assessment Strategy (pre/post; within/between** | **Type of Outcome Measure** | **Outcome Assessment Time Frame** | **Sig Increase in Empathy?** |
| --- | --- | --- | --- | --- | --- | --- | --- | --- | --- | --- |
| Daeppen et al., 2012 [53] | Medical Students | 91 | Yes | Yes | Motivational Interviewing (MI) | 8 | Post; between-group | Other-report | 1 week post-intervention | Yes |
| Delvaux et al., 2005 [54] | Physicians | 62 | Yes | Yes | Other | 18 | Pre & post; within & between-group | Other-report | Immediately following intervention | Yes |
| Dicki et al., 2009 [55] | Medical Students | 60 | No | N/A | Other | N/E | Pre & post; within-group | Other-report | Immediately following intervention; 6 months post | Yes |
| Dow et al., 2007 [56] | Residents | 20 | Yes | No | Other | 6 | Pre & post; within and between-group | Other-report | N/E | Yes |
| Fallowfield et al., 2002 [57] | Physicians | 160 | Yes | Yes | Other | N/E | Pre & post; between-group | Other-report | 3 months post-intervention | Yes |
| Farnill et al., 1997 [58] | Medical Students | 56 | No | N/A | Other | 16 | Pre & post; within-group | Self-report question | N/E | Yes |
| Fernandez-Olano et al., 2008 [59] | Medical Students & Residents | 203 | Yes | No | Communication Skills Training | 25 | Pre & post; within-group | Self-report survey | N/E | Yes |
| Fine & Therrien, 1977 [60] | Medical Students | 43 | Yes | No | Role Playing | 12 | Pre & post; within & between-group | Other-report | Immediately following intervention | Yes |
| Garcia et al., 2013 [61] | Physicians | 13 | No | No | Communication Skills Training | 14 | Pre & post; within-group | Other-report | 0-3 months post-intervention | Yes |
| Ghetti et al., 2009 [62] | Residents | 17 | No | N/A | Balint Training | 12 | Pre & post; within-group | Self-report survey | N/E | No |

N/A, Not Applicable; N/E, Not Explicitly Stated

**Table 1. Intervention studies evaluating quantitative changes in empathy.**

| **Article** | **Source Population** | **Sample Size** | **Control Group** | **Random Assignment** | **Intervention Type** | **Duration of Intervention (hours)** | **Assessment Strategy (pre/post; within/between)** | **Type of Outcome Measure** | **Outcome Assessment Time Frame** | **Sig Increase in Empathy?** |
| --- | --- | --- | --- | --- | --- | --- | --- | --- | --- | --- |
| Harlak et al., 2008 [63] | Medical Students | 59 | No | N/A | Communication Skills Training | 30 | Pre & post; within-group | Self-report survey | N/E | Yes |
| Hart et al., 2006 [64] | Residents | 28 | No | N/A | Other | 1-1.5 | Pre & post; within-group | Other-report | N/E | No |
| Hojat et al., 2013 [65] | Medical Students | 248 | Yes | Yes | Lecture & workshop | N/E | Pre & post; within & between-group | Self-report survey | Immediately following intervention; 10 weeks follow-up | Yes |
| Jenkins & Followfield, 2002 [66] | Physicians | 93 | Yes | Yes | Communication Skills Training | N/E | Pre & post; between-group | Other-report | 3 months post-intervention | Yes |
| Karaoglu & Seker, 2011 [67] | Medical Students | 195 | No | N/A | Problem Based Learning (PBL) | N/E | Pre & post; within-group | Self-report survey | Immediately following intervention | No |
| Kramer et al., 1989 [68] | Medical Students | 40 | Yes | Yes | Communication Skills Training | 15 | Pre & post; within & between-group | Other-report | 1 week, 6 months, 12 months post-intervention | Yes |
| Krasner et al., 2009 [69] | Physicians | 70 | No | N/A | Other | 52 | Pre & post; within-group | Self-report survey | Immediately following intervention; 2, 12, & 15 months post-intervention | Yes |
| Kushner et al., 2014 [70] | Medical Students | 127 | No | No | Communication Skills Training | N/E | Pre & post; within-group | Self-report survey | Immediately following intervention; 1 year post-intervention | Yes |
| Lienard et al., 2010 [71] | Residents | 98 | Yes | Yes | Communication Skills Training | 40 | Pre & post; between-group | Other-report | Immediately following intervention | Yes |
| Lienard et al., 2010 [72] | Residents | 88 | Yes | Yes | Communication Skills Training | 40 | Pre & post; between-group | Other-report | Immediately following intervention | No |
| Lim et al., 2011 [73] | Medical Students | 149 | Yes | No | Role Playing | 1 | Pre & post; within & between-group | Self-report survey | Immediately following intervention | Yes |

N/A, Not Applicable; N/E, Not Explicitly Stated

**Table 1. Intervention studies evaluating quantitative changes in empathy.**

| **Article** | **Source Population** | **Sample Size** | **Control Group** | **Random Assignment** | **Intervention Type** | **Duration of Intervention (hours)** | **Assessment Strategy (pre/post; within/between)** | **Type of Outcome Measure** | **Outcome Assessment Time Frame** | **Sig Increase in Empathy?** |
| --- | --- | --- | --- | --- | --- | --- | --- | --- | --- | --- |
| Misra-Hebert et al., 2012 [74] | Physicians | 36 | Yes | No | Humanities | 16 | Pre & post; between-group | Self-report survey | Immediately following intervention | Yes |
| Mitchell et al., 2011 [75] | Physicians | 13 | No | N/A | Motivational Interviewing (MI) | 8-10 | Pre & post; within-group | Other-report | N/E | Yes |
| Norfolk et al., 2009 [76] | Physicians | 24 | Yes | No | Other | N/E | Pre & post; within & between-group | Other-report | Immediately following intervention | Mixed |
| Ozcan et al., 2012 [77] | Medical Students | 143 | No | N/A | Communication Skills Training | 10 | Pre & post; within-group | Self-report survey | N/E | Yes |
| Pacala et al., 1995 [78] | Medical Students | 55 | Yes | No | Role Playing | 3 | Pre & post; within & between-group | Self-report survey | 1-2 weeks post-intervention | Yes |
| Poole & Sanson-Fisher, 1980 [79] | Medical Students | 45 | Yes | Yes | Communication Skills Training | 12-16 | Pre & post; within & between-group | Other-report | Immediately following intervention; 3 years post | Yes |
| Razavi et al., 2003 [80] | Physicians | 59 | Yes | Yes | Other | 37 | Pre & post; within & between-group | Other-report | N/E | Yes |
| Riess et al., 2011 [81] | Residents | 11 | No | N/A | Other | 4.5 | Pre & post; within-group | Self, other, patient-report | Immediately following intervention | Mixed |
| Riess et al., 2012 [82] | Residents & Fellows | 99 | Yes | Yes | Other | 3 | Pre & post; between-group | Self, other, patient-report | 1-2 months post-intervention | Mixed |

N/A, Not Applicable; N/E, Not Explicitly Stated

**Table 1. Intervention studies evaluating quantitative changes in empathy.**

| **Article** | **Source Population** | **Sample Size** | **Control Group** | **Random Assignment** | **Intervention Type** | **Duration of Intervention (hours)** | **Assessment Strategy (pre/post; within/between)** | **Type of Outcome Measure** | **Outcome Assessment Time Frame** | **Sig Increase in Empathy?** |
| --- | --- | --- | --- | --- | --- | --- | --- | --- | --- | --- |
| Rosenthal et al., 2011 [83] | Medical Students | 162 | No | N/A | Other | N/E | Pre & post; within-group | Self-report survey | N/E | No |
| Roter et al., 1995 [84] | Physicians | 69 | Yes | Yes | Communication Skills Training | 8 | Post; between-group | Other-report | N/E | Yes |
| Roter et al., 2004 [85] | Residents | 28 | No | N/A | Communication Skills Training | 4 | Pre & post; within-group | Other-report | N/E | Yes |
| Sands et al., 2008 [86] | Physicians | 19 | No | N/A | Humanities | 6 | Pre & post; within-group | Self-report survey | Immediately following intervention | No |
| Sanson-Fisher & Poole, 1978 [87] | Medical Students | 135 | Yes | No | Other | 12-16 | Pre & post; within & between-group | Other-report | N/E | Mixed |
| Sanson-Fisher & Poole, 1980 [88] | Medical Students | 40 | No | N/A | Communication Skills Training | N/E | Pre & post; within-group | Other-report | N/E | No |
| Schell et al., 2013 [89] | Fellows | 22 | No | N/A | Communication Skills Training | N/E | Pre & post; within-group | Self-report survey | Immediately following intervention | Yes |
| Scholer et al., 2008 [90] | Medical Students & Residents | 124 | No | N/A | Other | 0.67 (40 minutes) | Pre & post; within-group | Other-report | Immediately following intervention | Yes |
| Schweller et al., 2014 [91] | Medical Students | 247 | No | No | Other | N/E | Pre & post; within-group | Self-report survey | Immediately following intervention | Yes |
| Shapiro et al., 1998 [92] | Medical Students | 78 | Yes | Yes | Mindfulness Based Stress Reduction (MBSR) | 17.5 | Pre & post; within & between-group | Self-report survey | Immediately following intervention | Yes |

N/A, Not Applicable; N/E, Not Explicitly Stated

**Table 1. Intervention studies evaluating quantitative changes in empathy.**

| **Article** | **Source Population** | **Sample Size** | **Control Group** | **Random Assignment** | **Intervention Type** | **Duration of Intervention (hours)** | **Assessment Strategy (pre/post; within/between)** | **Type of Outcome Measure** | **Outcome Assessment Time Frame** | **Sig Increase in Empathy?** |
| --- | --- | --- | --- | --- | --- | --- | --- | --- | --- | --- |
| Shapiro et al., 2004 [93] | Medical Students | 16 | Yes | Yes | Humanities | 8 | Pre & post; within-group | Self-report survey | N/E | Mixed |
| Shapiro et al., 2006 [94] | Medical Students | 92 | Yes | Yes | Humanities | N/E | Post; between-group | Other-report | N/E | Mixed |
| Shapiro et al., 2009 [95] | Medical Students | 79 | Yes | Yes | Communication Skills Training | N/E | Pre & post; between-group | Other-report | 4 months post-intervention | Yes |
| Smith et al., 1995 [96] | Residents | 26 | Yes | Yes | Other | N/E | Pre & post; between-group | Patient-report survey | N/E | No |
| Sripada et al., 2011 [97] | Residents | 12 | Yes | Yes | Other | N/E | Pre & post; between-group | Self-report, patient-report | Immediately following intervention | Yes |
| Tiuraniemi et al., 2011 [98] | Medical Students | 126 | No | N/A | Other | 6 | Pre & post; within-group | Self-report survey | Immediately following intervention | No |
| Tulsky et al., 2011 [99] | Physicians | 48 | Yes | Yes | Communication Skills Training | N/E | Post; between-group | Other-report, patient-report | Immediately following intervention; 1 week post | Yes |
| Van Winkle et al., 2012 [100] | Medical Students | 183 | No | N/A | Humanities | 0.67 (40 minutes) | Pre & post; within-group | Self-report survey | Immediately following intervention; 26 days post | Yes |
| Varkey et al., 2006 [101] | Medical Students | 84 | No | N/A | Role Playing | 3 | Pre & post; within-group | Self-report survey | Immediately following intervention | Yes |

N/A, Not Applicable; N/E, Not Explicitly Stated

**Table 1. Intervention studies evaluating quantitative changes in empathy.**

| **Article** | **Source Population** | **Sample Size** | **Control Group** | **Random Assignment** | **Intervention Type** | **Duration of Intervention (hours)** | **Assessment Strategy (pre/post; within/between)** | **Type of Outcome Measure** | **Outcome Assessment Time Frame** | **Sig Increase in Empathy?** |
| --- | --- | --- | --- | --- | --- | --- | --- | --- | --- | --- |
| Walters et al., 2007 [102] | Physicians | 22 | No | N/A | Role Playing | N/E | Pre & post; within-group | Other-report | 1 month post-intervention | Yes |
| Winefield & Chur-Hansen, 2000 [103] | Medical Students | 115 | No | N/A | Communication Skills Training | 3 | Pre & post; within-group | Other-report | Immediately following intervention | Yes |
| Wolf et al., 1987 [104] | Medical Students | 223 | Yes | Yes | Other | 12 | Pre & post; within & between-group | Other-report | Immediately following intervention | Yes |
| Yang et al, 2013 [105] | Medical Students & Residents | 110 | No | No | Other | 4 | Pre & post; within-group | Self-report survey | Immediately following intervention | No |

N/A, Not Applicable; N/E, Not Explicitly Stated
