# Supplementary material for: Interventions to cultivate physician empathy: a systematic review
Source: BMC Med Educ. 2014 Oct 14;14:219. doi: 10.1186/1472-6920-14-219 (PMC4201694; doi:10.1186/1472-6920-14-219)
Supplement: Supplementary file 3 — Additional file 3: Table S2: Quality assessment of intervention studies. Description of Dataset: Table showing the quality assessment results of all 64 studies. (DOCX 114 KB) [file 12909_2013_1041_MOESM3_ESM.docx]

**Table 2. Quality assessment of intervention studies.**

| **Article** | **Control Group** | **Random Assignment** | **Outcome Measure** | **Study Design Quality** | **Sig Increase in Empathy?** | **Sample Size** |
| --- | --- | --- | --- | --- | --- | --- |
| Airagnes et al., 2014 [42] | Yes | No | + | Tier 2 | No | 439 |
| Bayne, 2011 [43] | No | N/A | + | Tier 3 | Yes | 22 |
| Bays et al., 2014 [44] | No | No | - | Tier 3 | Yes | 128 |
| Bond et al., 2013 [45] | No | N/A | + | Tier 3 | No | 27 |
| Bonvicini et al.,  2009 [46] | Yes | Yes | +/- | Tier 2 | Yes | 114 |
| Bosse et al., 2012 [47] | Yes | Yes | +/- | Tier 2 | Yes | 92 |
| Bunn & Terpstra, 2009 [48] | Yes | Yes | + | Tier 1 | Yes | 150 |
| Cahan et al., 2010 [49] | Yes (pilot 1); No (pilot 2) | No (pilot 1); N/A (pilot 2) | - | Tier 3 | Mixed | 97 (pilot 1), 44 (pilot 2) |
| Cataldo et al., 2005 [50] | Yes | No | + | Tier 2 | No | 114 |
| Chunharas et al., 2013 [51] | Yes | Yes | - | Tier 3 | Mixed | 89 |
| Cinar et al., 2012 [52] | No | N/A | +/- | Tier 3 | No | 20 |
| Daeppen et al., 2012 [53] | Yes | Yes | + | Tier 1 | Yes | 91 |
| Delvaux et al., 2005 [54] | Yes | Yes | - | Tier 3 | Yes | 62 |
| Dicki et al., 2009 [55] | No | N/A | - | Tier 3 | Yes | 60 |
| Dow et al., 2007 [56] | Yes | No | - | Tier 3 | Yes | 20 |

(+) Reliable and valid outcome measure; (+/-) Reliable, but not valid outcome measure; (-) Neither reliable, nor valid outcome measure. Tier 1: Control group, random assignment, reliable and valid outcome measure; Tier 2: Control group, no random assignment, reliable and valid outcome measure, or, Control group, random assignment, reliable but not valid outcome measure; Tier 3: All other study designs.

**Table 2. Quality assessment of intervention studies.**

| **Article** | **Control Group** | **Random Assignment** | **Outcome Measure** | **Study Design Quality** | **Sig Increase in Empathy?** | **Sample Size** |
| --- | --- | --- | --- | --- | --- | --- |
| Fallowfield et al., 2002 [57] | Yes | Yes | + | Tier 1 | Yes | 160 |
| Farnill et al., 1997 [58] | No | N/A | - | Tier 3 | Yes | 56 |
| Fernandez-Olanao et al., 2008 [59] | Yes | No | + | Tier 2 | Yes | 203 |
| Fine & Therrien, 1977 [60] | Yes | No | - | Tier 3 | Yes | 43 |
| Garcia et al., 2013 [61] | No | No | - | Tier 3 | Yes | 13 |
| Ghetti et al., 2009 [62] | No | N/A | + | Tier 3 | No | 17 |
| Harlak et al., 2008 [63] | No | N/A | + | Tier 3 | Yes | 59 |
| Hart et al., 2006 [64] | No | N/A | + | Tier 3 | No | 28 |
| Hojat et al., 2013 [65] | Yes | Yes | + | Tier 1 | Yes | 248 |
| Jenkins & Fallowfield, 2002 [66] | Yes | Yes | + | Tier 1 | Yes | 93 |
| Karaoglu & Seker, 2011 [67] | No | N/A | + | Tier 3 | No | 195 |
| Kramer et al., 1989 [68] | Yes | Yes | - | Tier 3 | Yes | 40 |
| Krasner et al., 2009 [69] | No | N/A | + | Tier 3 | Yes | 70 |
| Kushner et al., 2014 [70] | No | No | - | Tier 3 | Yes | 127 |
| Lienard et al., 2010 [71] | Yes | Yes | - | Tier 3 | Yes | 98 |

(+) Reliable and valid outcome measure; (+/-) Reliable, but not valid outcome measure; (-) Neither reliable, nor valid outcome measure. Tier 1: Control group, random assignment, reliable and valid outcome measure; Tier 2: Control group, no random assignment, reliable and valid outcome measure, or, Control group, random assignment, reliable but not valid outcome measure; Tier 3: All other study designs.

**Table 2. Quality assessment of intervention studies.**

| **Article** | **Control Group** | **Random Assignment** | **Outcome Measure** | **Study Design Quality** | **Sig Increase in Empathy?** | **Sample Size** |
| --- | --- | --- | --- | --- | --- | --- |
| Lienard et al., 2010 [72] | Yes | Yes | - | Tier 3 | No | 88 |
| Lim et al., 2011 [73] | Yes | No | + | Tier 2 | Yes | 149 |
| Misra-Hebert et  al., 2012 [74] | Yes | No | + | Tier 2 | Yes | 36 |
| Mitchell et al., 2011 [75] | No | N/A | + | Tier 3 | Yes | 13 |
| Norfolk et al., 2009 [76] | Yes | No | +/- | Tier 3 | Mixed | 24 |
| Ozcan et al., 2012 [77] | No | N/A | + | Tier 3 | Yes | 143 |
| Pacala et al., 1995 [78] | Yes | No | - | Tier 3 | Yes | 55 |
| Poole & Sanson-Fisher, 1980 [79] | Yes | Yes | - | Tier 3 | Yes | 45 |
| Razavi et al., 2003 [80] | Yes | Yes | - | Tier 3 | Yes | 59 |
| Riess et al., 2011 [81] | No | N/A | + | Tier 3 | Mixed | 11 |
| Riess et al., 2012 [82] | Yes | Yes | + | Tier 1 | Mixed | 99 |
| Rosenthal et al., 2011 [83] | No | N/A | + | Tier 3 | No | 162 |
| Roter et al., 1995 [84] | Yes | Yes | - | Tier 3 | Yes | 69 |

(+) Reliable and valid outcome measure; (+/-) Reliable, but not valid outcome measure; (-) Neither reliable, nor valid outcome measure. Tier 1: Control group, random assignment, reliable and valid outcome measure; Tier 2: Control group, no random assignment, reliable and valid outcome measure, or, Control group, random assignment, reliable but not valid outcome measure; Tier 3: All other study designs.

**Table 2. Quality assessment of intervention studies.**

| **Article** | **Control Group** | **Random Assignment** | **Outcome Measure** | **Study Design Quality** | **Sig Increase in Empathy?** | **Sample Size** |
| --- | --- | --- | --- | --- | --- | --- |
| Roter et al., 2004 [85] | No | N/A | + | Tier 3 | Yes | 28 |
| Sands et al., 2008 [86] | No | N/A | + | Tier 3 | No | 19 |
| Sanson-Fisher & Poole, 1978 [87] | Yes | No | - | Tier 3 | Mixed | 135 |
| Sanson-Fisher & Poole, 1980 [88] | No | N/A | - | Tier 3 | No | 40 |
| Schell et al., 2013 [89] | No | N/A | - | Tier 3 | Yes | 22 |
| Scholer et al., 2008 [90] | No | N/A | - | Tier 3 | Yes | 124 |
| Schweller et al., 2014 [91] | No | No | + | Tier 3 | Yes | 247 |
| Shapiro et al., 1998 [92] | Yes | Yes | +/- | Tier 2 | Yes | 78 |
| Shapiro et al., 2004 [93] | Yes | Yes | + | Tier 1 | Mixed | 16 |
| Shapiro et al., 2006 [94] | Yes | Yes | - | Tier 3 | Mixed | 92 |
| Shapiro et al., 2009 [95] | Yes | Yes | + | Tier 1 | Yes | 79 |
| Smith et al., 1995 [96] | Yes | Yes | +/- | Tier 2 | No | 26 |
| Sripada et al., 2011 [97] | Yes | Yes | + | Tier 1 | Yes | 12 |
| Tiuraniemi et al., 2011 [98] | No | N/A | +/- | Tier 3 | No | 126 |

(+) Reliable and valid outcome measure; (+/-) Reliable, but not valid outcome measure; (-) Neither reliable, nor valid outcome measure. Tier 1: Control group, random assignment, reliable and valid outcome measure; Tier 2: Control group, no random assignment, reliable and valid outcome measure, or, Control group, random assignment, reliable but not valid outcome measure; Tier 3: All other study designs.

**Table 2. Quality assessment of intervention studies.**

| **Article** | **Control Group** | **Random Assignment** | **Outcome Measure** | **Study Design Quality** | **Sig Increase in Empathy?** | **Sample Size** |
| --- | --- | --- | --- | --- | --- | --- |
| Tulsky et al., 2011 [98] | Yes | Yes | + | Tier 1 | Yes | 48 |
| Van Winkle et al., 2012 [100] | No | N/A | + | Tier 3 | Yes | 183 |
| Varkey et al., 2006 [101] | No | N/A | - | Tier 3 | Yes | 84 |
| Walters et al., 2007 [102] | No | N/A | - | Tier 3 | Yes | 22 |
| Winefield & Chur-Hansen, 2000 [103] | No | N/A | +/- | Tier 3 | Yes | 115 |
| Wolf et al., 1987 [104] | Yes | Yes | - | Tier 3 | Yes | 223 |
| Yang et al., 2013 [105] | No | No | + | Tier 3 | No | 110 |

(+) Reliable and valid outcome measure; (+/-) Reliable, but not valid outcome measure; (-) Neither reliable, nor valid outcome measure. Tier 1: Control group, random assignment, reliable and valid outcome measure; Tier 2: Control group, no random assignment, reliable and valid outcome measure, or, Control group, random assignment, reliable but not valid outcome measure; Tier 3: All other study designs.
