# Supplementary figures and images for: Interventions to cultivate physician empathy: a systematic review
Source: BMC Med Educ. 2014 Oct 14;14:219. doi: 10.1186/1472-6920-14-219 (PMC4201694; doi:10.1186/1472-6920-14-219)

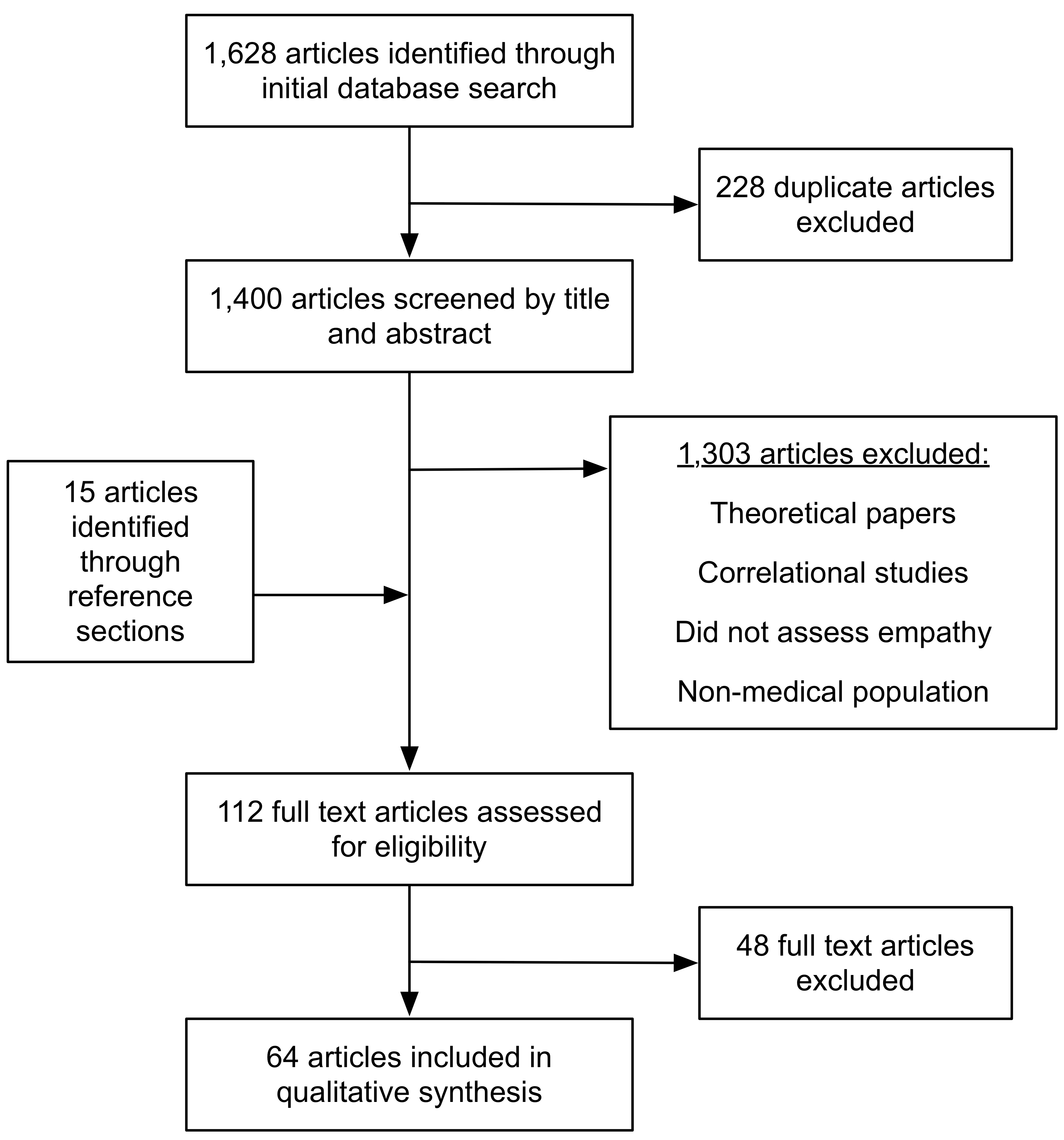

Supplement: Supplementary file 4 — Authors’ original file for figure 1 [file 12909_2013_1041_MOESM4_ESM.tiff]

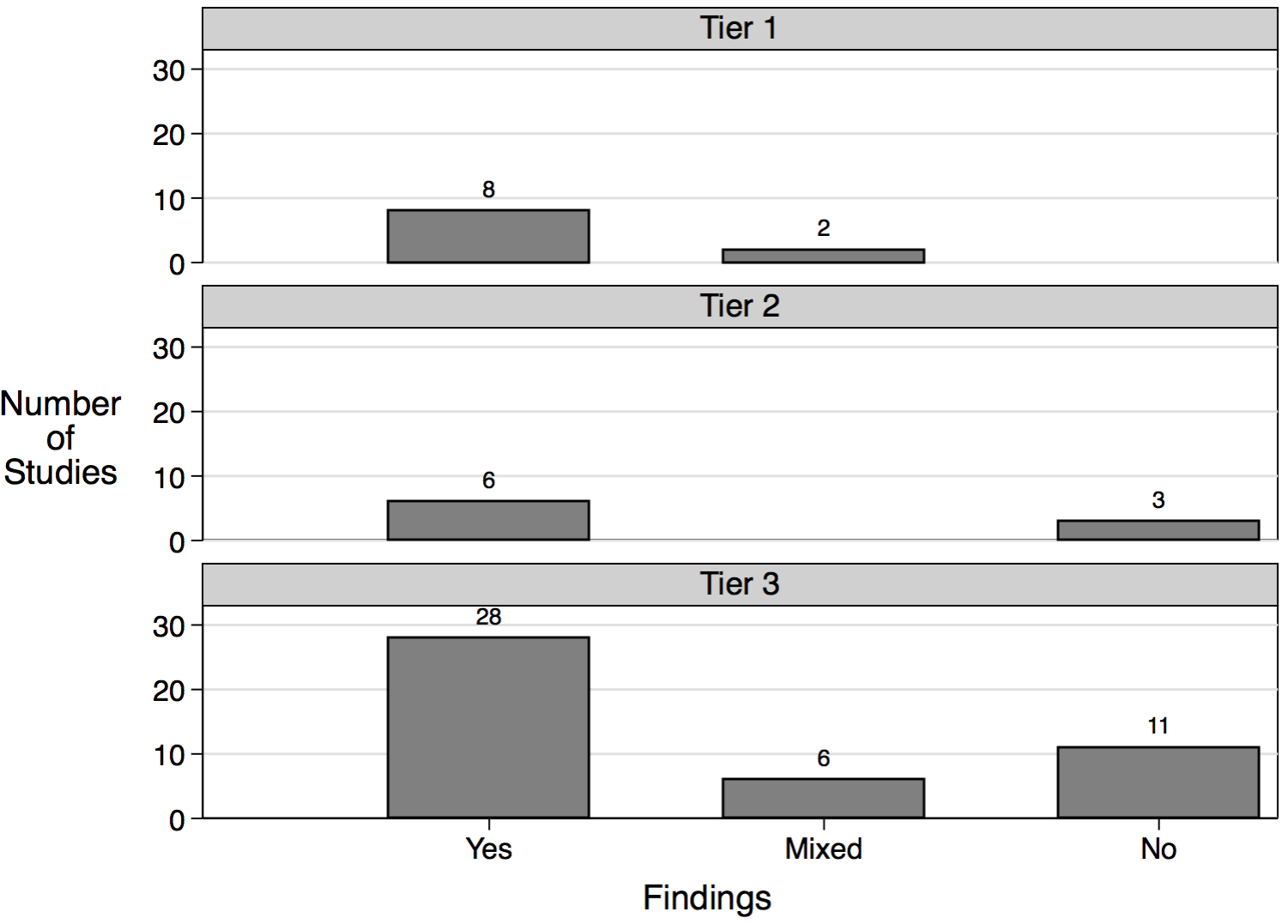

Supplement: Supplementary file 5 — Authors’ original file for figure 2 [file 12909_2013_1041_MOESM5_ESM.tiff]
